# Invasive *Microascus trigonosporus* Species Complex Pulmonary Infection in a Lung Transplant Recipient

**DOI:** 10.1155/2015/745638

**Published:** 2015-05-14

**Authors:** Kelly E. Schoeppler, Martin R. Zamora, Noelle M. Northcutt, Gerard R. Barber, Gayle O'Malley-Schroeder, Dennis M. Lyu

**Affiliations:** ^1^Department of Pharmacy, University of Colorado Hospital, Aurora, CO 80010, USA; ^2^Department of Pulmonary and Critical Care Medicine, University of Colorado Anschutz Medical Campus, Aurora, CO 80010, USA; ^3^Hospitalist Medicine, Denver Health and Hospital, Denver, CO 80204, USA; ^4^Department of Mycology, University of Colorado Anschutz Medical Campus, Aurora, CO 80010, USA

## Abstract

Because of the high incidence of morbidity and mortality associated with invasive fungal infections, antifungal prophylaxis is often used in solid organ transplant recipients. However, this prophylaxis is not universally effective and may contribute to the selection of emerging, resistant pathogens. Here we present a rare case of invasive infection caused by *Microascus trigonosporus* species complex in a human, which developed during voriconazole prophylaxis in a lung transplant recipient. Nebulized liposomal amphotericin B was used in addition to systemic therapy in order to optimize antifungal drug exposure; this regimen appeared to reduce the patient's fungal burden. Despite this apparent improvement, the patient's pulmonary status progressively declined in the setting of multiple comorbidities, ultimately leading to respiratory failure and death.

## 1. Introduction

Invasive fungal infections, most commonly with* Candida* spp. and* Aspergillus* spp., are associated with a high morbidity and mortality in immunocompromised hosts [1]. For this reason, antifungal prophylaxis active against these pathogens is often employed. However, antifungal prophylaxis is not universally effective and may contribute to the selection of emerging, resistant pathogens [2]. Here we present a rare case of invasive infection caused by* Microascus trigonosporus* species complex in a human, which developed during voriconazole prophylaxis.

## 2. Case Report

A 64-year-old male underwent bilateral lung transplantation at an outside hospital in 2011 for idiopathic pulmonary fibrosis with pulmonary hypertension. His comorbidities at the time of transplant were notable for New York Heart Association Class III Heart Failure with implantable cardioverter defibrillator placement. His postoperative course was complicated by severe primary graft dysfunction (PGD 3), acute rejection, renal failure requiring hemodialysis, tracheostomy placement,* Clostridium difficile* colitis and ileitis, cytomegaloviral viremia, bilateral upper extremity deep venous thrombi, pleurocutaneous fistula of the right chest necessitating pleurodesis, atrial fibrillation, and a gastrointestinal bleed. His postoperative course spanned six weeks in the intensive care unit. At hospital discharge, his forced expiratory volume in one second (FEV_1_) was 1.31 L/s or 43% of predicted.

**Figure 1:**
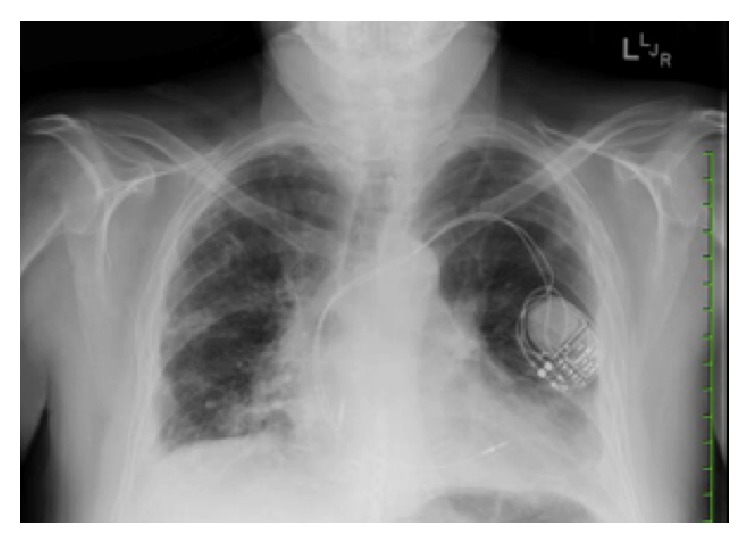
Chest radiography (CXR) showed diffuse patchy infiltrates and small bilateral pleural effusions.

**Figure 2:**
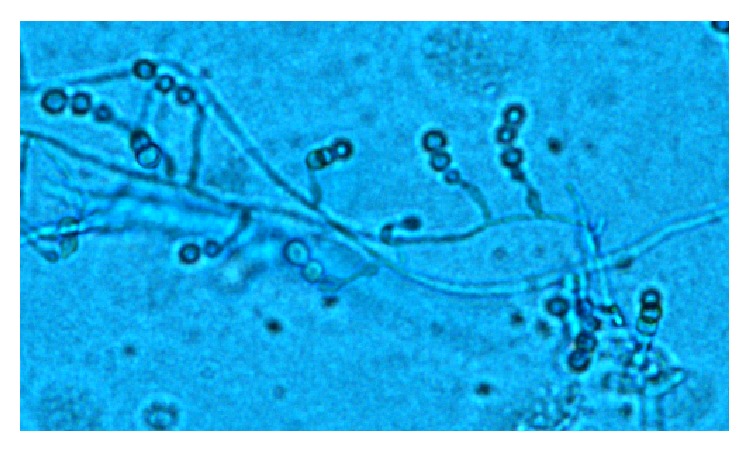
Microscopy. Lactophenol cotton blue stain. Note many overlapping conidiophores and conidia of* Scopulariopsis* sp. grown from patient's BAL sample fluid (10x).

Three months after discharge, he presented to our facility in follow-up after moving to our region. His immunosuppression consisted of tacrolimus (trough = 6.4 ng/mL), mycophenolate mofetil 250 mg twice daily, prednisone 10 mg daily, and inhaled fluticasone/salmeterol 250/50 mcg twice daily. He had been receiving voriconazole 200 mg twice daily for fungal prophylaxis since day 1 postoperatively in addition to sulfamethoxazole-trimethoprim and valganciclovir. At presentation he was noted to have worsening dyspnea, dry cough, increasing malaise, and low-grade fevers. Physical exam findings were consistent with distal airway narrowing by auscultation. FEV_1_ was 0.92 L/s (29% predicted). Chest radiography (CXR) showed diffuse patchy infiltrates and small bilateral pleural effusions (Figure 1). Bronchoscopy revealed multiple endobronchial strictures but no endobronchial plaques, and intravenous (IV) high-dose steroids (methylprednisolone 10 mg/kg once daily × 3 days) were given for probable acute rejection (AR). Transbronchial biopsies did not demonstrate AR (A0), but the bronchoalveolar lavage (BAL) fluid grew moderate mold 5 days later. Posaconazole 400 mg twice daily was started for empiric treatment of a breakthrough fungal infection, and the patient's esomeprazole was held to facilitate absorption. Speciation of the mold occurred at day 11, revealing moderate* Scopulariopsis* sp. on culture, with morphology most consistent with* S. brumptii* by microscopy (Figure 2). Later, DNA sequencing of the internal transcribed spacer (ITS) region would identify the mold as* Microascus trigonosporus* species complex. Confirmatory DNA sequencing of the organism was attempted at a second laboratory and was unsuccessful.

**Figure 3:**
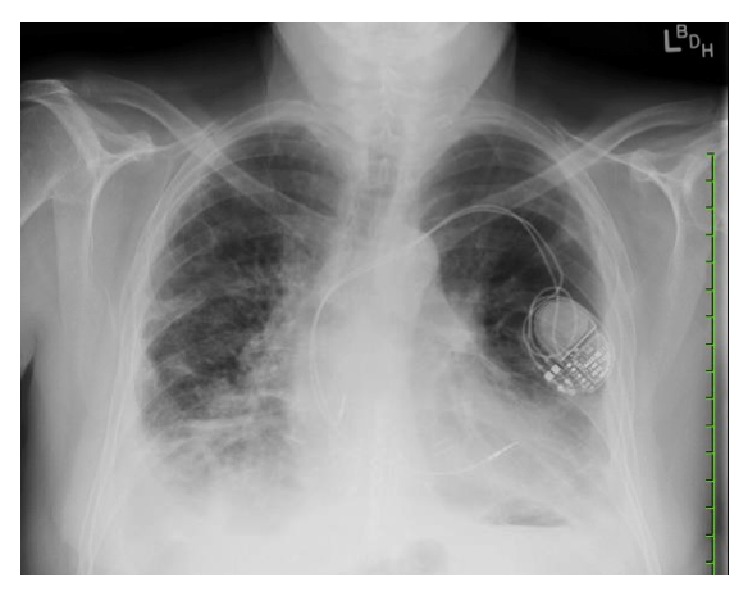
Chest radiography after two weeks of antifungal therapy showing right lower lobe opacity.

**Figure 4:**
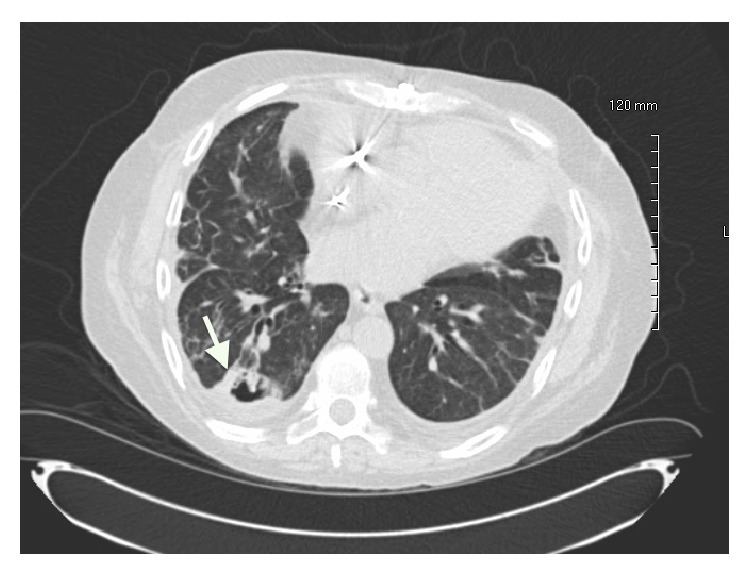
Axial chest computed tomography scan after two weeks of antifungal therapy showing thick-walled, subpleural cavitation and nodules (arrow).

**Figure 5:**
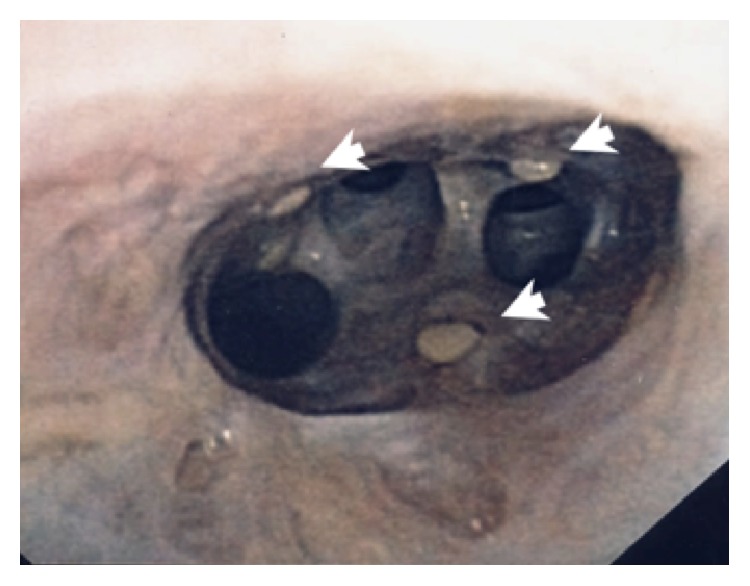
Bronchoscopy images after two weeks of antifungal therapy with diffusely abnormal appearing mucosa and tan adherent plaques (arrows).

**Figure 6:**
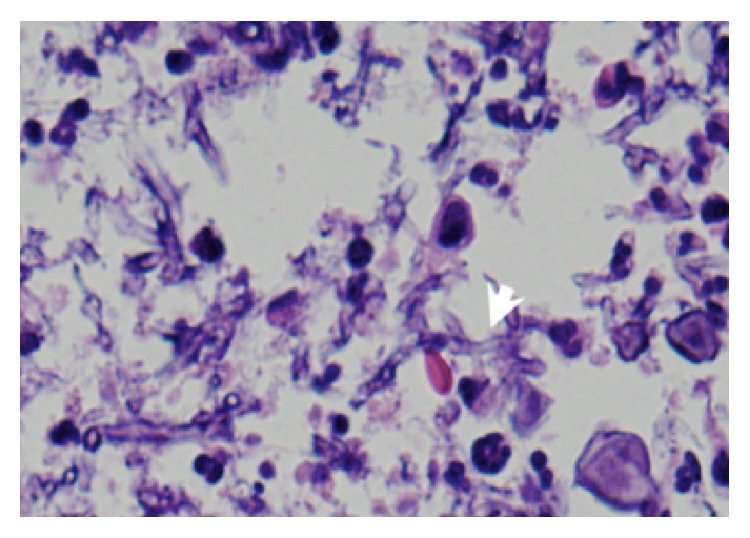
BAL cytology. H&E stain (high power, zoom) showing fungus hyphae which were hyaline and septated with 45-degree branching (arrow).

Following two weeks of posaconazole therapy, repeat CXR denoted increased right-sided pleural effusion and new infiltrates in the right lower lobe (Figure 3). FEV_1_ was 1.08 L/s (33% predicted). A chest computed tomography scan was notable for a thick-walled cavitary lesion with mural nodularity involving the subpleural posterior right lower lobe and tree-in-bud centrilobular nodules within the right lower lobe (Figure 4). Repeat bronchoscopy demonstrated endobronchial, adherent tan-colored plaques throughout, tan secretions, and diffusely abnormal appearing mucosa (Figure 5). Pathology results from a transbronchial biopsy of the cavitary lesion demonstrated septated fungal hyphae, consistent with possible* Aspergillus* spp. Endobronchial biopsies of the plaques were notable for the finding of a small, detached fragment of matted fungal hyphae adjacent to but not penetrating the endobronchial mucosa (Figure 6). The BAL fluid became positive at day 4 after biopsy for moderate mold, again identified as* Scopulariopsis* sp. The patient was deemed not to be a surgical candidate for resection of the mycetoma based on his comorbidities. He was admitted for treatment of his invasive fungal infection with IV liposomal amphotericin B.

Treatment with liposomal amphotericin B IV 4 mg/kg intravenously every 24 hours was initiated. In order to maximize antifungal drug concentrations at the site of the infection, liposomal amphotericin B 25 mg via nebulizer was given every 24 hours for five days and then reduced to three times a week. Posaconazole was also continued. Inhaled fluticasone/salmeterol and mycophenolate mofetil were held given the patient's active fungal infection. The patient was discharged on hospital day 8 in stable condition.

Three weeks after therapy was started, the patient presented to the hospital with hypoxemic respiratory failure with increased oxygen requirement. CXR was notable for worsened parenchymal opacities reflecting edema or pneumonia. The patient was admitted and treated with IV furosemide and an increased dose of liposomal amphotericin B IV (5 mg/kg daily). The patient developed respiratory failure requiring intubation and developed difficult to control atrial flutter with a rapid ventricular rate. Repeat bronchoscopy demonstrated a markedly lower volume of tan secretions, and only a single endobronchial plaque in the left mainstem bronchus was identified. However, the patient's pulmonary status continued to decline secondary to chronic rejection, cardiac dysrhythmia with pulmonary edema, and pulmonary infection. On the 11th day of hospitalization, the patient's advance directive to withdraw care resulted in death. His medical power of attorney refused autopsy on his behalf.

Further workup of the* Microascus trigonosporus* species complex later revealed the following minimum inhibitory concentrations (MICs, mcg/mL) obtained by the Clinical and Laboratory Standards Institute (CLSI) broth microdilution method: amphotericin B ≥ 8, itraconazole ≥ 16, posaconazole = 2, and voriconazole = 2. Minimum effective concentration (MEC, mcg/mL) for the echinocandins was also obtained: micafungin = 2, anidulafungin = 2, and caspofungin = 2.

## 3. Discussion

Here we report a rare case of invasive infection with* Microascus trigonosporus* species complex in a lung transplant recipient. DNA sequencing of the cultured organism proved necessary to distinguish clearer identification of the pathogen having a clinical appearance similar to more common pathogens in the lung transplant patient such as* Aspergillus* and a morphologic appearance similar to other species of* Scopulariopsis* (teleomorph* Microascus*).

Fungi of the genus* Scopulariopsis* are asexual and filamentous; their teleomorphs,* Microascus*, are also included in the genus. The fungi grow worldwide and are known to inhabit soil, plant material, insects, and other material.* Scopulariopsis* and* Microascus* spp. are more frequently the cause of superficial infections, including onychomycosis and keratitis [3]. Rarely, invasive disease caused by this genus of fungi has been described, including endocarditis, brain abscess, cutaneous infections, and localized and disseminated pulmonary infections [3–6]. Four* Scopulariopsis* species (*S. acremonium, S. brevicaulis, S. brumptii*,* and S. candida*) and two* Microascus* species (*M. cirrosus and M. cinereus*) have been definitively identified and reported as causing invasive human infection [3]. One reported case of* Microascus trigonosporus* pneumonia exists in the literature; however, this case did not describe evidence of the fungus on tissue biopsy but rather identified the organism based on morphology from a positive culture from the BAL fluid (nonsterile fluid) [7]. Confirmation test via DNA sequencing was not performed.

Invasive disease caused by* Microascus* and* Scopulariopsis* spp. primarily affects immunocompromised hosts and is associated with a high mortality in this population [3]. A paucity of data exists describing the susceptibility profile of* Microascus* and* Scopulariopsis* spp. In vitro studies have indicated that MICs of fluconazole, itraconazole, and flucytosine against* Scopulariopsis* spp. are high, and those of amphotericin B, voriconazole, and ketoconazole are variable [8–13]. Case reports also describe high-level resistance of* S. brevicaulis* and* S. acremonium* to amphotericin B, which correlated with poor clinical outcomes [14–16].

Because we knew little about the susceptibility pattern of the organism we were treating prior to obtaining the MICs postmortem other than its growth despite voriconazole prophylaxis, we used a combination of inhaled and systemic liposomal amphotericin B. In a study of inhaled liposomal amphotericin B for* Aspergillus* prophylaxis in lung transplant recipients, drug concentrations measured between the segmental bronchus and the parenchyma at 2, 7, and 14 days after nebulization were shown to exceed the MICs for the large majority of* Aspergillus* isolates [17, 18]. No evidence of lipid pneumonitis was found [17]. The addition of nebulized liposomal amphotericin B in this patient offered direct airway delivery of the antifungal, which allowed for an increase in total amphotericin B exposure without a high risk of additive systemic toxicity. This regimen appeared to reduce the endobronchial fungal burden despite high amphotericin MICs. Furthermore, the use of nebulized liposomal amphotericin B allowed for a convenient transition of care to the outpatient setting, as the pharmacokinetic properties of liposomal amphotericin B allow for three times weekly administration [17].

Identification of rare pathogenic molds infecting human hosts is imperative both for the optimization of treatment in the affected patient and for advancing our understanding in order to improve treatment strategies for future patients. Identification of molds based on phenotypic criteria alone can be flawed, and molecular methods of mold identification are the gold standard for species identification [19, 20]. DNA sequencing of the organism in this case was performed at two distinct clinical laboratories, and, despite this, definitive identification of the species was not possible. Therefore, this case is unique not only because of the rarity of the organism involved and the innovative treatment strategy that was utilized but also because it demonstrates the inherent difficulties in identification of rare molds that remain despite the recent advancements of molecular methods. The nucleotide sequence of the case isolate was deposited in the GenBank database under accession number HQ676488.

## 4. Conclusion 

Here we report a rare case of invasive infection with* Microascus trigonosporus* species complex in a human. It developed in a lung transplant recipient despite antifungal prophylaxis with voriconazole. Nebulized liposomal amphotericin B was used in addition to systemic therapy in order to optimize drug exposure; this regimen appeared to reduce the patient's fungal burden. Despite this, this patient's* Microascus trigonosporus* species complex infection likely contributed to his worsening pulmonary status, which ultimately led to his death.
